# Pilot study of online ante-mortem inspection for emergency slaughtered cattle in Denmark

**DOI:** 10.3389/fvets.2025.1570452

**Published:** 2025-04-02

**Authors:** Maybritt Kiel Poulsen, Lis Alban, Annette Dresling

**Affiliations:** ^1^Department of Food and Veterinary Issues, Danish Agriculture and Food Council, Copenhagen, Denmark; ^2^Department of Veterinary and Animal Sciences, University of Copenhagen, Frederiksberg, Denmark

**Keywords:** risk-based inspection, remote inspection, bovines, videorecording, sensing technology

## Abstract

According to the EU legislation, bovines subjected to emergency slaughter must undergo a physical ante-mortem inspection (AMI) on the farm before slaughter if the meat is to be placed on the market for human consumption. This pilot study examined the performance of online AMI using a software system that enabled a video consultation between the veterinarian and the farmer. During 2022–2023, 38 bovines, possibly eligible for emergency slaughter, were included in the study. A comparison between online and physical AMI was carried out. Furthermore, a comparison was made between two different veterinarians (vet.1 and vet.2) about the performance of AMI for each bovine. The results showed that vet.1 considered the online judgement as adequate for 95% of the cases in the study, whereas vet.2 considered 90% adequate. Moreover, the agreement between the two veterinarians was high (accuracy = 90%), both regarding the adequacy of the system and the eligibility of the bovine for slaughter. Prerequisites for using online AMI are suitable video equipment, a reliable internet connection and sufficient light. Furthermore, the use of a checklist will help to assure standardized online AMI. Saving the recorded videos will reduce the risk of fraud and enable training and calibration of veterinarians. In conclusion, online AMI of bovines destined for emergency slaughter will, in most cases, be comparable to physical AMI on-farm. However, the use of online AMI presupposes a change of the EU legislation.

## 1 Introduction

Official meat inspection is a prerequisite for evaluating whether meat from food-producing animals can be placed on the market for human consumption. The procedure consists of ante-mortem inspection (AMI) of live animals and post-mortem inspection (PMI) of carcasses and organs. Typically, both activities are performed by an official veterinarian (OV) on-site at the abattoir, but it is also possible to perform AMI on-farm, e.g., in cases of emergency slaughter. Both AMI and PMI focus on the verification of animal welfare, animal health and food safety, as specified in Article 17 of the EU Regulation 2017/625 (1).

In Denmark, approximately 450,000 cattle are slaughtered annually (2), and 3,300 of these (0.7%) are subjected to emergency slaughter (own data). The Danish Veterinary and Food Administration (DVFA) has specified that an animal eligible for emergency slaughter is an otherwise healthy animal that has suffered an acute injury, e.g., a limb fracture or a hip luxation (3). After stunning and exsanguination, the carcass is transported to the abattoir for further processing, including PMI.

Formerly, the private practicing veterinarian was able to perform the AMI of bovines that were potentially eligible for emergency slaughter. In 2019, this practice was amended when the EU legislation was updated as written in Article 4 of the EU Regulation 2019/624 (4). Since then, it has been a requirement that the AMI is performed by an OV. Furthermore, the OV must now observe that the stunning and exsanguination of the bovine is performed correctly. The way this was implemented in Denmark was associated with high administrative costs, resulting in higher expenses than formerly. This created an incentive for the farmer to send the animal for rendering, which is inconsistent with a more sustainable approach to meat production.

According to Article 16, Point 2b in EU Regulation 2017/625, the EU Commission is obliged to consider scientific and technical developments in meat inspection when adopting new legislation (1). Hence, for every new technology, there is a need to investigate its performance. The aim of this pilot study was, therefore, to investigate the performance of online AMI, consisting of a video consultation with pictures and sound between the farmer and the veterinarian. To assess the performance, the adequacy and eligibility were investigated, where adequacy referred to whether the veterinarian could see what they are required to during the AMI. In contrast, eligibility dealt with whether a bovine could be directed to emergency slaughter instead of rendering. The specific objectives were to investigate:

1. The adequacy of online AMI
2. The judgement of eligibility for emergency slaughter
3. The agreement between two different veterinarians’ judgement
4. The adequacy of using online AMI to judge stunning and exsanguination
5. The strengths, weaknesses, opportunities and threats (SWOT) associated with using online AMI of bovines that are potentially eligible for emergency slaughter

## 2 Materials and methods

### 2.1 Study design

The study participants consisted of veterinarians from a Danish cattle practice and their approximately 160 cattle farmers, located in Southern Jutland. Seven of the cattle veterinarians in the practice were appointed by the DVFA as OVs, which allowed them to perform AMI of emergency slaughtered bovines, and three of the seven participated in the study. Each month, the veterinarians attend 15–25 bovines that are potentially eligible for emergency slaughter (personal communication PV Hansen, Ribe Veterinary Practice, 2022). It was the intention to include data from 50 such bovines. This figure was judged as sufficiently large to reveal relevant information for the pilot study. The data were collected from 1 June 2022 to 31 December 2023. Before the start, the three cattle veterinarians were trained in using the chosen software system. Moreover, a short Q&A about the study was developed to support the dialogue between the veterinarians and their farmers. The involved farmers were informed about the study when they contacted the veterinary practice about an emergency slaughter or when the veterinarian was visiting the farm for other reasons. The procedure used when a cattle farmer agreed to participate in the study is described in Annex 1 in the Supplementary material.

A software solution created by the Danish company, Incendium,^1^ was used. This software was designed to find, in the geographical area concerned, the optimal internet connection available between the veterinarian (vet.1) and the farmer. It enabled vet.1 to establish a video connection with the farmer using a mobile phone. In total, the online AMI included (1) two recordings, one of vet.1 and one of the farmer with the bovine, and (2) the dialogue between the farmer and vet.1. The farmer filmed the areas on the animal when directed to do so by vet.1, and the recordings were saved on the server belonging to Incendium.

**Figure 1:**
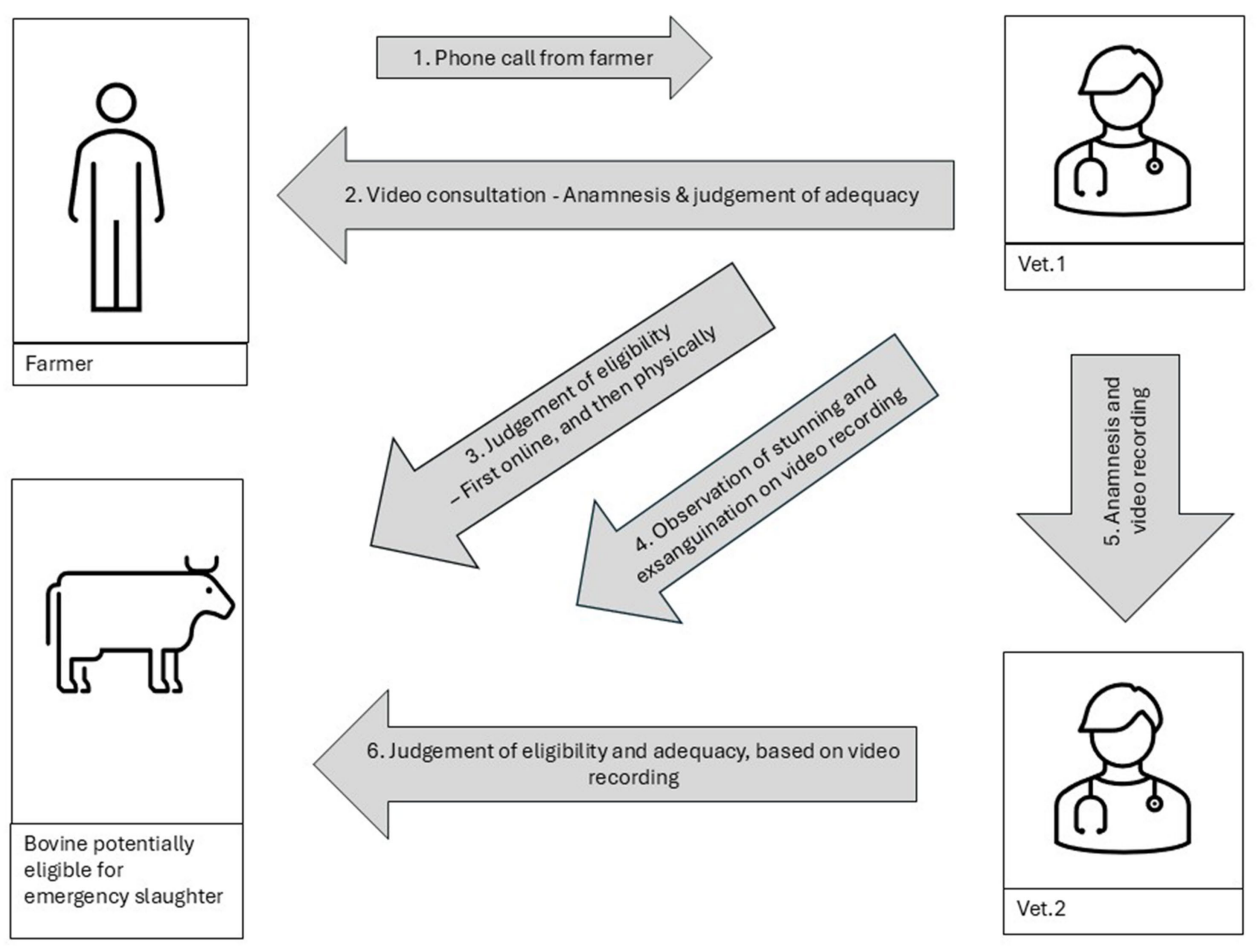
Graphical display of the applied study design.

The study design is depicted in Figure 1. It was designed so vet.1, after collecting the anamnesis, first performed the online AMI and then went to the farm to perform the physical AMI, since it is not currently a legal option to perform online AMI. When vet.1 performed the online AMI, vet.1 acted as a private practitioner, but when vet.1 performed the physical AMI on-farm, vet.1 acted as an OV. A predefined electronic checklist was used to help ensure uniform clinical examinations during the different online AMIs of the study bovines (Annex 2 in the Supplementary material). The checklist covered information about the farmer, identification number of the bovine, anamnesis, time and place of the AMI and all relevant clinical observations. First, vet.1 noted whether an online approach could be used to undertake AMI (adequacy), and next, whether vet.1 found the bovine fit for emergency slaughter (eligibility). After the subsequent physical AMI in the herd, vet.1 used the same form to record if findings were observed during the physical AMI that were not detectable during the online AMI. Vet.1 also noted whether these findings were considered to have an impact on the evaluation of the bovine as eligible or not for emergency slaughter. The electronic checklist was then saved in the software system. If possible, stunning and exsanguination of the bovine were recorded on video using a mobile phone.

Next, the video recording and the anamnesis of the bovine was shown to another cattle veterinarian (vet.2). Vet.2 assessed whether they considered the online AMI as adequate and whether the video recording was sufficient to determine the bovine’s eligibility for emergency slaughter. Vet.2 was not informed about the observations and conclusions made by vet.1. The criterion for acting as vet.2 was that they had not been acting as vet.1 in the same case. A predefined electronic checklist, resembling the one used by vet.1, was used by vet.2 to ensure their uniform evaluation (Annex 3 in the Supplementary material). If the bovine was found eligible for emergency slaughter, data from the subsequent PMI of that animal were collected from the Danish cattle slaughter database (own data).

To quantify the level of agreement between the two veterinarians’ judgements, the accuracy was calculated as the proportion of cases where vet.1 and vet.2 agreed. Next, Cohen’s Kappa (5) and the prevalence-and bias-adjusted kappa (PABAK) statistics (6) were calculated using the epi.kappa function from the epiR package in R (7). The input data, structured as a 2×2 matrix of frequencies, were analysed with a 95% confidence level and a two-sided alternative hypothesis.

The pilot study was continuously evaluated throughout the study period at meetings attended by the study coordinators, the involved veterinarians and Incendium to ensure optimal technique, compliance with the inclusion and exclusion criteria and other adjustments in relation to unforeseen events. Based on these discussions, it was possible to retrieve information for an informal analysis of the strengths, weaknesses, opportunities and threats (SWOT) associated with online AMI using video recordings. Only recordings where the SMS-to-video technique functioned properly, and the sound and images were clear, were included in the study. Moreover, an inclusion criterion was that the recorded dialogue between the farmer and the veterinarian included information about the anamnesis and clinical information about the bovine.

## 3 Results

The data collection ran from 1 June 2022 to 31 December 2023. The raw data included 55 recordings, with most lasting between 1.5 to 2 min. Subsequently, twelve were excluded due to technical issues. In six of these, the sound was absent or poor. Three only included a recording of either vet.1 or the farmer, and in the remaining three, the pictures were blurred and pixelated. Hence, a total of 43 recordings met the inclusion criteria. Unfortunately, in five of these, mistakes were made when filling in the checklist or the evaluation form, whereby these had to be discarded. Therefore, the subsequent analysis included recordings of the remaining 38 bovines. The most frequent anamneses were various limb injuries caused by slipping, or hip dislocation in connection with parturition. The animals slipped, e.g., when walking on wet, slatted floors, bumping into stable fixtures or when mounting or being mounted by other animals in heat (Table 1).

**Table 1:** Anamnesis reported for 38 Danish bovines included in a study on online ante-mortem inspection of animals for emergency slaughter.

Anamnesis	Number of bovines (%)
Hip dislocation	22 (57.9)
Luxation/tendon injury	4 (10.5)
Fracture	3 (7.9)
Wound	1 (2.6)
Paresis	1 (2.6)
Other acute injuries	7 (18.4)

The results showed that the online judgement was considered adequate by both two veterinarians in most cases (vet.1: 36/38 = 94.7% and vet.2: 34/38 = 89.5%). The overall agreement between vet.1 and vet.2 regarding adequacy of using online AMI was high for accuracy (89.5%), while kappa was fair (0.283) and PABAK was high (0.789) (Table 2). In four out of 38 cases (10%), the two veterinarians disagreed about the adequacy. The disagreements dealt with the quality of the recording (*n* = 3) and unavailable temperature of the animal (*n* = 1) (Annex 4 in the Supplementary material). Moreover, agreement between the two veterinarians regarding eligibility of the bovines for slaughter was very high for accuracy (89.5%), while kappa was moderate (0.539), and PABAK was high (0.789) (Table 2). In 31 out of 38 cases (82%), vet.1. and vet.2 agreed that the bovine in question was eligible for emergency slaughter, and in three cases (8%) they agreed that the bovine should be sent for rendering,

**Table 2:** Association between two veterinarians, called vet.1 and vet.2, regarding the adequacy of using online AMI and the judgement of eligibility for emergency slaughter of 38 Danish bovines.

Vet.1 Online AMI	Vet.2 Online AMI		Total	Kappa	PABAK
Adequacy	Adequate	Inadequate			
Adequate	33 (87%)	3 (8%)	36 (95%)	0.283	0.789
Inadequate	1 (3%)	1 (3%)	2 (5%)		
Total	34 (90%)	4 (10%)	38 (100%)		
Eligibility	Eligible	Ineligible	Total		
Eligible	31 (82%)	2 (5%)	33 (87%)	0.539	0.789
Ineligible	2 (5%)	3 (8%)	5 (13%)		
Total	33 (87%)	5 (13%)	38 (100%)		

Vet.1. Online AMI	Vet.1 Physical AMI		Total	Kappa	PABAK
Eligibility	Eligible	Ineligible			
Eligible	32 (84%)	1^a^ (3%)	33 (87%)	0.445	0.789
Ineligible	3^b^ (8%)	2 (5%)	5 (13%)		
Total	35	3	38 (100%)		

^aNo. 54. bNo. 20, No. 52 and No. 55.^

In four of the 38 cases, vet.1 made additional findings during the physical AMI which resulted in a change of the judgement of the bovine (Annex 5 in the Supplementary material). If only online AMI had been used, three bovines eligible for slaughter would have been sent for rendering and one bovine that should have been rendered would have been slaughtered (Table 2). So, the agreement between vet.1’s online and physical judgement was high for accuracy (89.5%), while kappa was moderate (0.445%) and PABAK was high (0.789) (Table 2). In total, three animals were euthanized on the farm and sent for rendering, and one animal was slaughtered on-farm for the farmer’s own use. Moreover, 34 bovines were emergency slaughtered on the farm and sent to an abattoir for further processing. All were unconditionally approved after PMI at the abattoir (own data).

Finally, six recordings of the stunning and exsanguination show that the person performing the stunning waited until the bovine stood still and did not move the head. Then, the captive bolt gun was placed on the forehead and fired. Before sticking, animal-based indicators were used to ensure that the bovine was stunned, i.e., collapsed and with an absence of corneal reflex. The results of the SWOT analysis provide an overview of the main issues that should preferably be considered before implementing online AMI (Table 3).

**Table 3:** Results of SWOT analysis regarding use of online AMI of bovines in relation to emergency slaughter.

Strengths	Weaknesses
Better animal welfare, because the animal will be inspected sooner Can be used in areas not easily accessible due to geography or bad weather conditions Improved sustainability through prevention of unnecessary food waste and fewer transport resources for the OV Increased flexibility for OV and farmer OV can drive to the farm if in doubt about the online AMI Checklist ensures a standardized approach Calibration of OV assessments through training and education	Requires (1) good internet connection, (2) equipment like a Smartphone or similar devices, (3) suitable light conditions and (4) training/information of farmers and veterinarians ” The sense” of the animal including smell and touch is missing The system must be set up to avoid fraud Should not be used in areas with outbreaks of notifiable and contagious animal diseases Reluctance to use system (conservatism) Legislation must be changed

Opportunities	Threats
Method is applicable for AMI in other situations (slaughter on-farm) Further development of software solutions is expected in the future People’s positive experience could result in the use of other online solutions in the agricultural sector	Risk of fraud with animals and data Legislation remains unchanged, so online AMI cannot be used Symptoms of contagious livestock diseases could be overlooked

## 4 Discussion

The data collection was initially scheduled from 1 June 2022 to 31 December 2022, but due to unforeseen challenges, the data collection period was prolonged and finalised 31 December 2023. The reasons were start-up problems using the IT system and farmers being busy during the harvest seasons, and therefore, unwilling to participate in the study. Furthermore, several bovines eligible for emergency slaughter were inspected when the veterinarian was already visiting the farm for another reason and could therefore not be included in our study.

In most cases where vet.1 and vet.2 disagreed whether online AMI was adequate, the reasons were technical, like poor internet connection or insufficient light, which resulted in a poor-quality recording, making it difficult to assess the bovine in question. Hence, sufficient light and internet connection are essential when assessing a bovine online.

According to Daniel et al. (8) a fast and stable internet connection is a prerequisite for using remote meat inspection. The software we used was designed to find the optimal internet connection at a given location, and which enabled recordings of both vet.1 and the farmer; this software feature was crucial for our study. In Denmark, most areas have good network coverage, but there might be regions where the use of online AMI could be hampered. We suggest that the farmer is always asked about network coverage and sufficient light before the online AMI is initiated.

The high overall agreement for AMI judgements observed between vet.1 and vet.2 indicates that online AMI in most cases is comparable to a clinical examination of a bovine on-farm. A similar study conducted at low-capacity abattoirs in Sweden also found a commercially available software programme (using Facetime) useful for remote AMI of pigs, sheep and cattle (9). That study concluded that remote AMI is at least as capable as physical AMI to detect non-compliances in the animals. It should be noted that the Swedish study may not be directly comparable to the present study, as the Swedish study dealt with healthy animals, whereas ours dealt with injured animal possibly eligible for emergency slaughter. The sample size in our study is limited and included only bovines from clients using one veterinary practice. Still, we consider the collected information as sufficient to assess the performance of online AMI.

In four (3+1) cases, findings were made during the physical AMI that were not recognisable during the online AMI, and these findings altered the decision initially made by vet.1, based on the online AMI. This indicates that online AMI might not be applicable in all cases of emergency slaughter, but could constitute an alternative to physical AMI, since the assessment based on the online AMI was confirmed by the physical AMI in 92% of the cases. This result contrasts the abovementioned study by Kautto et al. (9), where remote AMI was shown to be more detailed than physical AMI. Moreover, in the current study, two animals would have been sent for rendering if online AMI only had been used, but the physical AMI showed these animals were, in fact, eligible for emergency slaughter. This indicates that unnecessary food waste in this part of the meat chain cannot be fully avoided using online AMI. Nonetheless, the opportunity to use this system would enable the use of many animals that would otherwise be sent for rendering when physical AMI is not possible or economically sustainable. Hence, the amount of unnecessary food waste would be reduced.

One animal (No. 54) was found eligible for emergency slaughter by both vet.1 and vet.2 based on online AMI, whereas the physical AMI resulted in the animal being sent for rendering. This is, of course, an undesirable scenario. In comparison, data from the Danish cattle slaughter database show that only 36 (0.9%) out of 3,902 emergency slaughtered cattle in 2023 were condemned after PMI, whereas the rest (99.1%) were approved for human consumption. In our study, 34 of the 38 animals were emergency slaughtered on farm and sent to an abattoir for further processing, and all of these were subsequently approved for human consumption after PMI, according to relevant data from the Danish cattle slaughter database (own data). This indicates that cattle sent for emergency slaughter in Denmark are healthy and fit for slaughter in general, despite the acute injury that is the reason for the emergency slaughter. In a Swedish study about prerequisites and expectations for remote meat inspection (10), concerns were raised about the OV being able to address welfare issues efficiently at a distance. Hunka et al. (10) concluded that the OV always has the legal right to perform a physical inspection. Therefore, in case of doubt, the OV can drive to the abattoir and make sure that the right decisions are taken. The same approach should be used for online AMI.

If online AMI is allowed in the future, it would likely be used in all EU Member States. It might also include other domesticated species than bovines. To avoid spreading diseases and to prevent fraud, we suggest that online AMI should not be applied in regions, territories or countries with outbreaks of contagious animal diseases, such as foot-and-mouth disease, which is currently (January 2025) observed in Germany (11). Moreover, it would be relevant to save video recordings and checklists, because they could help prevent fraud. Additionally, the recordings could be used for training and calibration of veterinarians to ensure more precise and uniform decisions about AMI (9).

The average duration of the recordings was short, mostly 1.5–2 min, and it is not obvious from the dialogues with the farmers that vet.1 used the checklist in detail. From the dialogues it was apparent that the focus was on the injury of the bovine, if this could be located. Next, vet.1 usually asked to see the animal’s walk and gait, to evaluate its general condition, for the animal’s body temperature and then verified the ear tag number. The participating veterinarians were experienced cattle veterinarians, and they observed many of the information points on the checklist just by looking at the animal during the video consultation without involving the farmer directly. In most cases, the body temperature was measured by farm staff prior to the online AMI and then orally communicated to vet.1 during the online AMI. To avoid uncertainty, mistakes and the risk of fraud, we advise that the temperature measurement is carried out during the online AMI. A predesigned checklist covering all important issues will need to be used to ensure a standardised approach and before the veterinarian can evaluate the animal. This is in line with Almqvist et al. (12), who concluded that a prerequisite for remote PMI of pigs is a standardised method and sufficient inspection time. We suggest adding a question to the checklist about treatment with veterinary medicines. If the withdrawal period is not respected, the animal is unfit for consumption and must be condemned. The most frequent anamneses for the emergency slaughter in our study were various limb injuries caused by slipping or hip dislocation in connection with parturition. Experience shows that these kinds of injuries in general are easily recognizable by both farmers and veterinarians (13).

Initially, the participating veterinarians were sceptical about using a video consultation, because they were concerned about the lack of “the sense of the animal,” implying the smell and touch the veterinarians experience during the physical AMI. During the study, the veterinarians became more familiar with the system, which indicates that experience in using a remote video system for video consultations is essential. In line with this, Daniel et al. (8) concluded that the most important skill for the users of a digital device for remote meat inspection was the ability to interpret the incoming data correctly, and that the user must trust the incoming data is accurate and reliable. Focus on training in the use of new technologies is, therefore, of paramount importance, as also suggested by Grau-Noguer et al. (14).

The initial intention of the study was to record the stunning and exsanguination of each bovine that was emergency slaughtered. This was because, according to the legislation, the OV is obliged to observe that the stunning and exsanguination of the animal is performed correctly (15). Many farmers enrolled in our study found it unpleasant to film this. However, the six video recordings of these processes show it is possible to use a mobile phone to observe stunning and exsanguination. We believe that there is no need for additional data to describe this process, because stunning and exsanguination are carried out by persons with sufficient skills and a simple and uniform processes compared to online AMI. When done correctly, there are very few variations in the way the bolt gun can be handled and the animal exsanguinated. Usually, the animal is lying down, although sometimes it can be elevated for faster exsanguination. The use of animal-based welfare indicators was easily observed on the recordings, e.g., the absence of corneal reflex and collapse (16). Hence, observation of stunning and exsanguination should also be performed online, should online AMI be approved for use.

## 5 Conclusion

This pilot study indicates that, in most cases, online AMI is comparable to physical AMI on-farm. Before allowing the use of online AMI, prerequisites should be defined to ensure a uniform approach. Important prerequisites are (1) devices that can be used for a video consultation like Smartphones, (2) a stable and reliable internet connection, (3) sufficient light, and (4) training of the veterinarians and farmers in how to use the system. To ensure proper documentation, the recordings and filled-in checklists should be saved for further random checks.

## Data Availability

The raw data supporting the conclusions of this article will be made available by the authors, without undue reservation.

## Footnotes

^1^https://bliksund.com/ – later Bliksund.
