# Supplementary material for: Pilot study of online ante-mortem inspection for emergency slaughtered cattle in Denmark
Source: Front Vet Sci. 2025 Apr 2;12:1570452. doi: 10.3389/fvets.2025.1570452 (PMC11999972; doi:10.3389/fvets.2025.1570452)
Supplement: Supplementary file 1 [file Supplementary_file_1.docx]

# Supplementary materials

## Annex 1

**How the farmers consent was obtained**

The farmer received a SMS-to-video link from vet.1, if the farmer agreed to participate in the study. When the farmer activated the link, a short text appeared on the screen informing about the study and asking for consent by pressing “OK”. Then the video connection between the farmer and the veterinarian was initiated. After completion the farmer was automatically redirected to a special page on the DAFC website. Here, the farmer could read more about how the personal data were handled etc.

## Annex 2

**Checklist for veterinarian 1**

| **Online AMI of cattle potentially eligible for emergency slaughter** |
| --- |
| Date: Time period: Online □ |
| Name of the official veterinarian: |
| Herd identification number (CHR No.): |
| Animal identification number (CKR No.): |
| Person on-farm requesting the emergency slaughter: |
| Person on-farm participating in the AMI: |
| When was the injury discovered? (date/time) |
| Anamnesis/reason for emergency slaughter? |
| Description of the injury, if possible: |
| First-hand impression of the animal: |

**Clinical examination:**

| Parameters (mark with an X) | | Not possible to examine (NPE) or not relevant (NR) | Remarks |
| --- | --- | --- | --- |
| Movement | □ Normal  □ Abnormal (e.g., nervous behaviour)  □ Limping  □ Lying down |  |  |
| Eyes | □ Normal  □ Dehydrated  □ Pain |  |  |
| Nostrils | □ Moist  □ Moderately dry  □ Dry and dirty |  |  |
| Teeth grinding | □ Normal  □ Severe |  |  |
| Abdominal volume | □ Normal according to age/lactation  □ A little sunken  □ Very sunken |  |  |
| Hair coat | □ Shiny  □ Dull, bristly |  |  |
| Respiration | □ Slow < 10/min  □ Normal 10-30/min  □ Fast >30/min |  |  |
| Temperature | □ Low <38°C  □ Normal 38.0≥ and <38.8°C  □ Slightly raised 38.8< and ≤39°C  □ Fever > 39°C |  |  |
| Udder | □ Normal  □ Moderately asymmetrical  □ Severely swollen and discoloured |  |  |

Conclusion:

| **Remarks** |
| --- |
| Other remarks concerning the online AMI (e.g., lighting, technical issues, collaboration with the operator (filming with the mobile phone on farm etc.): |
| Is the animal eligible for emergency slaughter?  Yes/No |
| Is the online AMI sufficient to evaluate if the animal is eligible for emergency slaughter?  Yes/No |
| **After the physical AMI** |
| Were there findings during the physical AMI that were not seen during the online AMI?  If so, please describe. |
| Did these findings have an impact on whether the animal was found eligible for emergency slaughter?  If so, please describe |

## Annex 3

**Evaluation form for veterinarian 2**

| **Online AMI on farm of cattle potentially eligible for emergency slaughter** |
| --- |
| Your email address: |
| Name and authorisation number of the OV: |
| Herd identification number (CHR No.): |
| Animal identification number (CKR No.): |
| Date of evaluation: |
| Description of the injury, if possible: |
| First-hand impression of the animal: |

**Clinical examination:**

*Remark is obligatory

| Parameters (mark with an X) | | Not possible to examine (NPE) or not relevant (NR) | Remarks * |
| --- | --- | --- | --- |
| Movement | □ Normal  □ Abnormal (e.g. nervous behaviour)  □ Limping  □ Lying down  □ Not possible to examine (NPE) |  |  |
| Eyes | □ Normal  □ Dehydrated  □ Pain  □ Not possible to examine (NPE)  □ Not relevant (NR) |  |  |
| Nostrils | □ Moist  □ Moderately dry  □ Dry and dirty  □ Not possible to examine (NPE)  □ Not relevant (NR) |  |  |
| Teeth grinding | □ Normal  □ Severe  □ Not possible to examine (NPE)  □ Not relevant (NR) |  |  |
| Hair coat | □ Shiny  □ Dull, bristly  □ Not possible to examine (NPE)  □ Not relevant (NR) |  |  |
| Respiration | □ Slow < 10/min  □ Normal 10-30/min  □ Fast >30/min  □ Not possible to examine (NPE)  □ Not relevant (NR) |  |  |
| Temperature | Low <38°C  □ Normal 38.0≥ and <38.8°C  □ Slightly raised 38.8< and ≤39°C  □ Fever > 39°C □ Not possible to examine (NPE)  □ Not relevant (NR) |  |  |
| Udder | □ Normal  □ Moderately asymmetrical  □ Severely swollen and discoloured  □ Not possible to examine (NPE)  □ Not relevant (NR) |  |  |

Annex 4

The reason for disagreement in four cases where the two veterinarians (vet.1 and vet.2) disagreed on the adequacy of online AMI.

| Video number | Vet.1.: AMI adequate:  Yes/No | Vet.2.: AMI adequate: Yes/No | Reason for disagreement |
| --- | --- | --- | --- |
| 13 | Yes | No | Vet.2 found the video recording did not reveal the temperature and stated that online AMI could not be used. If temperature data had been available and if the temperature had been within the normal range, then vet.2 would have approved the bovine for emergency slaughter |
| 22 | Yes | No | Vet.2 found the video recording was too dark to evaluate the bovine properly and stated that online AMI could not be used |
| 50 | No | Yes | Vet.1 found the video recording was too pixelated and stated that online AMI could not be used |
| 52 | Yes | No | Vet.2 found the video recording was too blurred and stated that online AMI could not be used |

Annex 5

Findings at the physical AMI that altered the decision made by vet.1 using online AMI.

| Video  number | Important findings at physical AMI not observed at online AMI? | Online AMI could be used?  Yes/No | Online AMI  Eligible for slaughter:  Yes/No | Physical AMI  Eligible for slaughter:  Yes/No | Findings at the physical AMI |
| --- | --- | --- | --- | --- | --- |
| 20 | Yes | No | No | Yes | Vet.1 found online AMI inadequate for evaluating the bovine and found it ineligible for emergency slaughter. However, after the physical AMI the bovine was found eligible for slaughter, because the appearance and physical state of the bovine were directly related to the injury it had suffered. |
| 52 | Yes | Yes | No | Yes | Vet.1 found online AMI adequate for evaluating the bovine and found it ineligible for emergency slaughter. However, at the physical AMI the bovine was found eligible for emergency slaughter despite its low temperature. |
| 54 | Yes | Yes | Yes | No | Vet.1 found online AMI adequate for evaluating the bovine and found it eligible for emergency slaughter. However, at the  physical AMI the bovine appeared to be in a poor condition, it was cold, listless and generally affected with a high respiratory frequency not recognisable on video. |
| 55 | Nothing mentioned by mistake | Yes | No | Yes | Vet.1 failed to notice the additional findings at the physical AMI. |
